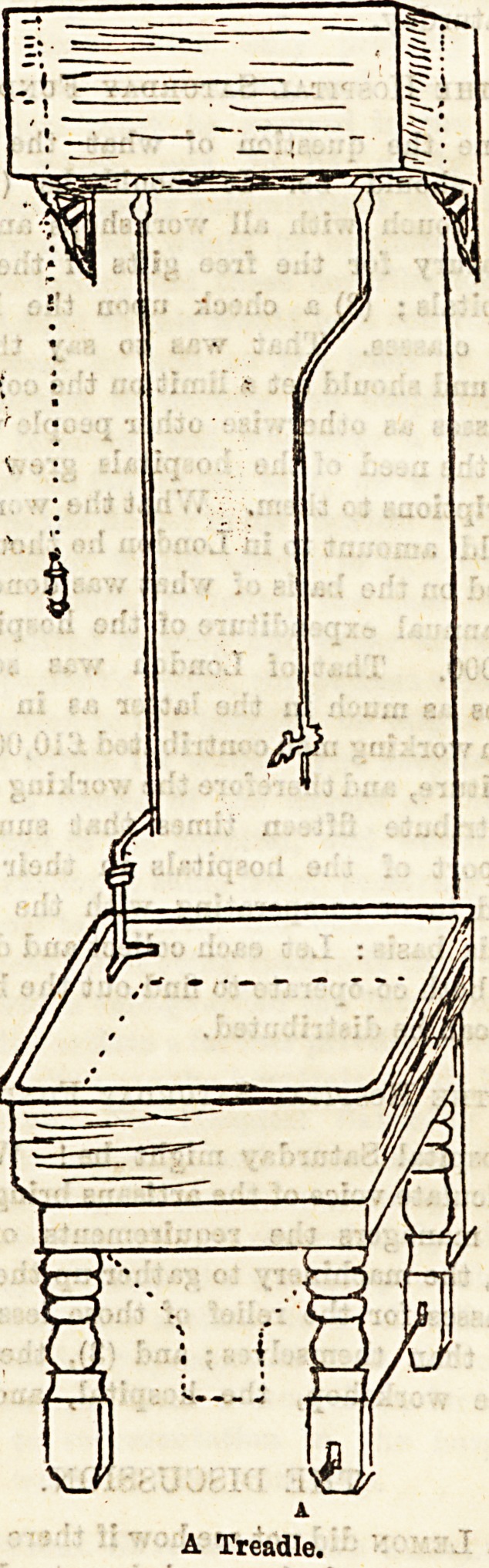# A Model Slop Sink

**Published:** 1892-05-07

**Authors:** 


### FURNITURE AND FITTINGS.

### A MODEL SLOP SINK.

An inspection of several institutions will reveal the fact that there are a variety of slop sinks in use, and that very few present any particular features or advantages. The illustration below represents an appliance wherein every detail has been carefully thought out for practical purposes. It is made of strong porcelain, about two feet square, almost unbreakable, and finished inside and over the rims with white enamel; a specially-made nozzle top is fixed over the centre fed from small cistern, the valve of which is opened by a treadle arrangement, so that the attendant can get the water required by placing the foot on the treadle, having both

A Treadle.

hands at liberty for other purposes. There is also a specially made brass flusher fixed at either corner of the sink supplied by ordinary syphon cistern, so that no time is lost by the attendant. This slop sink is in general use at the Royal Infirmary, Liverpool, and other large institutions, and has been supplied by Messrs. Gardner and Sons, of Duke Street, Liverpool. The cost of the sink, with pine frame, with ball top, treadle, brass flusher, and nozzle top, is £7 7s. An oak frame can be supplied at 10s. extra.

## Figures and Tables

**Figure f1:**